# Hydrocele Masking Testicular Tumour With Extensive Nodal Disease: A Case Report and Literature Review

**DOI:** 10.7759/cureus.43455

**Published:** 2023-08-14

**Authors:** Hani Sayedin, Ramandeep Chalokia, Rene Woderich

**Affiliations:** 1 Urology, Warrington and Halton Teaching Hospitals NHS Foundation Trust, Warrington, GBR

**Keywords:** delayed diagnosis, primary hydrocele, metastatic testicular cancer, scrotal lump, testicular swelling

## Abstract

Hydrocele is one of the most common causes of scrotal swellings. Fluid accumulation within the tunica vaginalis, a remnant of the peritoneum covering the testicle, leads to scrotal swelling. It is known to be a benign condition with no subsequent complications apart from increasing in size causing discomfort. Some patients could cope with the swelling effect and continue their life with no desire for further management while others are not fit for surgical intervention and would be treated conservatively with the same concept. However, once the testicle becomes swollen by the surrounding fluid, it would be difficult to examine the testicle itself even by an expert physician. We present here a 46-year-old patient who has been diagnosed with right hydrocele for a long time. The patient noticed general weakness and loss of weight. Initial investigations showed iron deficiency anemia and imaging showed retroperitoneal lymphadenopathy. Eventually, testicular ultrasonography showed a right testicular tumour that was masked by a hydrocele, resulting in delayed presentation of metastatic testicular cancer.

## Introduction

Hydrocele is a clinical condition resulting from fluid accumulation in the tunica vaginalis. In adults, primary hydrocele is mostly caused by a disturbed balance between fluid production and absorption within the tunica vaginalis itself [1]. It usually presents with slowly growing scrotal swelling and no diurnal size change, and on clinical examination, it shows transillumination of the examining torchlight. Secondary hydrocele could occur secondary to testicular pathology particularly inflammatory process and filariasis in endemic areas [2]. In addition, hydrocele was reported as a postoperative complication for inguinal herniotomies, ventriculoperitoneal shunts, and renal transplants [3]. Identification of the hydrocele type, primary or secondary, would be crucial in the management. Particularly, the primary type, unlike the secondary type, could be treated conservatively in selected patients.

## Case presentation

**Figure 1:**
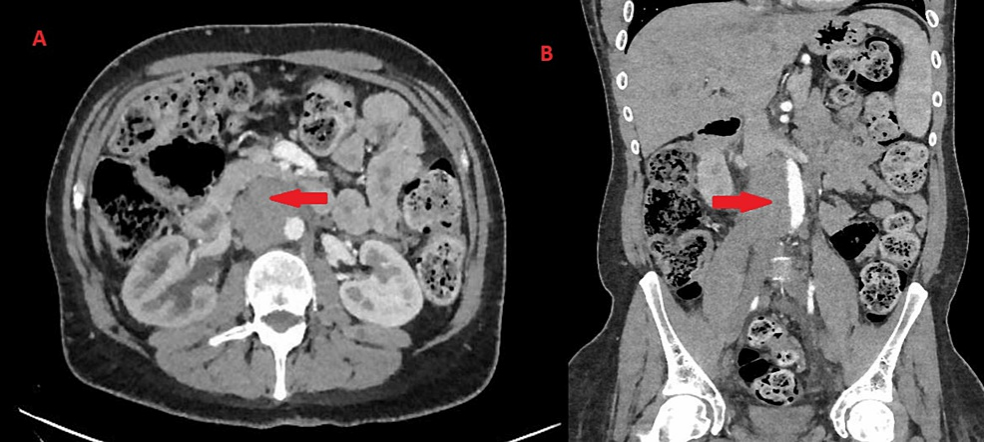
Contrast CT abdomen and pelvis Image "A" to the left is the axial view and shows para-aortic lymphadenopathy causing hydronephrosis. Image "B" to the right is the coronal view of the para-aortic nodal mass.

A 46-year-old man presented with loss of weight and general tiredness over three months. Initial investigations by a general practitioner revealed microcytic hypochromic anaemia (Hb 95 g/L (Ref. 130-180); MCV 81.7 (Ref. 85-105)). The patient's medical history included right hydrocele for the last eight years, diabetes mellitus, and schizophrenia. Therefore, the patient was referred initially to gastroenterology for assessment. A contrast CT study showed retroperitoneal para-aortic lymphadenopathy about 6 cm in longitudinal dimension with mild right hydronephrosis (Figure 1).

**Figure 2:**
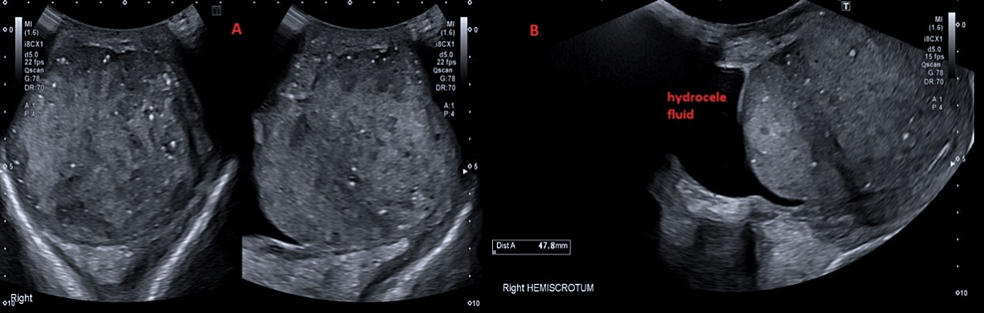
Testicular US Image "A" to the left is a transverse plane that shows an enlarged, heterogenous right testicle measuring 96 x 74 x 93 mm highly suspicious for malignancy. Image "B" to the right shows fluid collection/hydrocele in the right hemi-scrotum.

Lymphoma was one of the differential diagnoses. Therefore, the patient had a biopsy from the retroperitoneal lymph node, which showed a metastatic germ cell tumour. Therefore, testicular ultrasound was performed, and it showed right testicular enlargement, measuring 96 x 74 x 93 mm and heterogenous in echotexture, surrounded by fluid suggesting malignancy (Figure 2).

Therefore, the patient was referred to the urology department urgently, for assessment. Clinical examination showed tense cystic swelling in the right scrotum with a positive transillumination sign. Testicular tumour markers were requested, and alpha-fetoprotein (AFP) was normal (3.5 gm/nl (Ref. <7)). However, β human chorionic gonadotropin (β-HCG) was 4000 mLU/L (Ref. <2000) and lactic dehydrogenase (LDH) was 1309 U/L (Ref. 90-500). Subsequently, the patient underwent right inguinal orchidectomy, and histopathology confirmed seminoma invading the epididymis and the tunica vaginalis with lymphovascular invasion. A stage T2N3M0 S2, as per the tumour node metastasis (TNM) classification of the International Union Against Cancer (UICC), was diagnosed. Further chemotherapy was suggested by the oncology team, including four cycles of cisplatin and etoposide, over five days inpatient stay. Unfortunately, the patient died before starting his chemotherapy.

## Discussion

The testicle develops from the genital ridges, which are mesodermal in origin and lie within the abdominal cavity at the back of the foetus. From gestational weeks 10 to 15, testicular descent is guided by the gubernaculum to the scrotum under the influence of Mullerian inhibitory substance and insulin-like hormone 3 "relaxin-like factor" - transabdominal stage. After week 25, calcitonin gene-related polypeptide from the genitofemoral nerve acts on the gubernaculum to guide the testicular descent through the inguinal canal to the scrotum-inguinoscrotal stage [4]. During the testicular descent, the testicle takes its tunica - the tunica vaginalis - from the peritoneum. Then, the peritoneal canal connecting the testicle to the peritoneal cavity, called the processus vaginalis, undergoes a process of apoptosis that leads to obliteration [5]. Subsequently, failure of the process of obliteration leads to patency of the processus vaginalis, which leads to complications such as communicating hydrocele and inguinal hernia. In adults, non-communicating hydrocele would be more predominant. The tunica vaginalis of patients with hydrocele were compared to those of a non-hydrocele-infected male control. Aquaporin channel dysfunction, caused by overexpression of aquaporin channel one, could be linked to non-communicating hydrocele pathogenesis [6]. In addition, testicular organogenesis explains the blood supply and lymphatic drainage of the testicle. Gonadal arteries, which represent the main blood supply of the testicles, arise from the abdominal aorta at the L2 level. Therefore, the lymphatic supply follows the blood supply and the embryologic origin of the testicle; the inter-aortocaval lymph nodes for the right side and para-aortic lymph nodes for the left side [7].

Testicular tumours represent 1% of all male cancers and 5% of all urological tumours and are considered the most curable cancer. It is estimated that 470 cases would die of testicular cancer out of 9,190 new cases of testicular cancer in the USA in 2023 [8]. In 2022, the World Health Organization (WHO) classified testicular tumours as shown in Table 1.

**Table 1:** World Health Organization (WHO) classified testicular tumours in 2022 Source: [9]

Germ cell tumours derived from germ cell neoplasia in situ	Germ cell tumours unrelated to germ cell neoplasia in situ	Sex cord-stromal tumours of the testis	Ovarian-type tumours of the collecting ducts and rete testis	Ttumours of the collecting duct and rete testis	Para-testicular mesothelial tumours	Tumours of the epididymis	Metastatic tumours
Germ cell neoplasia in situ	Spermatocytic tumour Spermatocytic tumour with sarcomatous differentiation	Leydig cell tumour Leydig cell tumour. Malignant Leydig cell tumour.	Serous cystadenoma.	Adenoma of the collecting ducts and rete testis (adenoma)	Adenomatoid tumour	Cystadenoma of the epididymis	
Specific forms of intratubular germ cell neoplasia: Intratubular seminoma. Intratubular embryonal carcinoma. Intratubular trophoblast. Intratubular yolk sac tumour. Intratubular teratoma.	Teratoma, prepubertal type: Dermoid cyst. Epidermoid cyst.	Sertoli cell tumour (Sertoli cell tumours) Sertoli cell tumour. Malignant Sertoli cell tumour. Large cell calcifying Sertoli cell tumour.	Serous tumour of borderline malignancy (serous borderline tumour).	Adenocarcinoma of the collecting ducts and rete testis (adenocarcinoma)	Well-differentiated papillary mesothelial tumour	Papillary cystadenoma of the epididymis (papillary cystadenoma)	
Gonadoblastoma	Yolk sac tumour, prepubertal type	Granulosa cell tumours Adult granulosa cell tumour. Juvenile granulosa cell tumour.	Serous cystadenocarcinoma.		Mesothelioma Epithelioid mesothelioma. Sarcomatoid mesothelioma. Biphasic mesothelioma.	Adenocarcinoma of the epididymis	
Seminoma (the germinoma family of tumours)	Testicular neuroendocrine tumour, prepubertal type well-differentiated neuroendocrine tumour (monodermal teratoma)	Tumours in the fibroma thecoma group (the fibroma thecoma family of tumours) Thecoma. Fibroma.	Mucinous cystadenoma.			Squamous cell carcinoma of the epididymis (squamous cell carcinoma)	
Non-seminomatous germ cell tumours: Embryonal carcinoma. Yolk sac tumour, post-pubertal type. Choriocarcinoma. Placental site trophoblastic tumour. Epithelioid trophoblastic tumour. Cystic trophoblastic tumour. Teratoma, post-pubertal type. Teratoma with somatic type malignancy.	Mixed teratoma and yolk sac tumour, prepubertal type	Mixed and other sex cord-stromal tumours Mixed sex cord-stromal tumour. Signet ring stromal tumour. Myoid gonadal stromal tumour. Sex cord-stromal tumour, NOS.	Mucinous borderline tumour.			Melanotic neuroectodermal tumour of the epididymis (melanotic neuroectodermal tumour)	
Mixed germ cell tumours. Polyembryoma. Diffuse embryoma.			Mucinous cystadenocarcinoma.				
Germ cell tumours of unknown type. Regressed germ cell tumours			Endometrioid tumour, borderline.				
			Endometrioid adenocarcinoma.				
			Clear cell adenocarcinoma.				
			Brenner tumour.				

Testicular cancer is common among young men aged 15 to 45 years. The usual presentation of testicular tumours is a painless testicular lump. However, a painful testicular lump was prescribed in 5-10% of cases [10]. Kim YW et al. reported pancreatic cancer metastasis to the testicle, causing hydrocele, which was treated initially by a surgical approach [11]. Histopathological examination of the tunica revealed adenocarcinoma and further radiological investigations proved primary pancreatic tail cancer. Sakuma T et al. reported right hydrocele as a presentation for metastatic colon cancer that was confirmed after radical orchidectomy [12]. However, this patient was already known to have sigmoid cancer and was treated by colectomy 16 months prior to his presentation with testicular swelling. Ioannidis O et al. reported hydrocele as a presentation for anaplastic anaemia in a 36-year-old male patient [13]. The patient had a family history of anaplastic seminoma and hydrocele aspirate fluid cytology-confirmed malignant cells prior to surgical removal. Ben Kirdis et al. reported a granulosa cell tumour presented as a hydrocele [14]. Granulosa cell tumours are sex cord tumours, not germ cell neoplasia, which are not common (3 % of testicular tumours) and are associated with endocrinal abnormalities. For instance, the reported case showed gynecomastia, which paid attention to early assessment and further management by orchidectomy in an early stage prior to metastasis, unlike our case, which was a seminoma with no endocrinal abnormalities. Albino G et al. reported the incidental finding of paratesticular tumours during hydrocele operation [15]. The patient had hydrocele for 30 years and was assessed preoperatively by testicular US, which showed thickened tunica vaginalis. Surprisingly, a mass was found on exploration and the frozen section showed borderline papillary cystadenoma. Therefore, orchidectomy was performed and histopathological examination revealed a “papillary serous tumour of low malignant potential (PSTLMP)”; further imaging proved no metastasis.

Hydrocele might be associated with testicular cancer and masks its clinical presentation. Subsequently, malignancy might occur, resulting in delayed presentation or surprising intraoperative findings.

## Conclusions

A hydrocele is a benign scrotal condition, and the patient might be not encouraged for surgical intervention. However, testicular cancer usually presents with a lump in the scrotum. Therefore, a hydrocele might mask any underlying testicular malignancy. We believe that in case of delayed or abandoned surgical management of hydroceles, testicular ultrasound surveillance might help avoid missing the occurrence of malignancy to avoid the delayed presentation of testicular cancer. No specific guidance can be applied and more cases need to be reported.
